# Supplementary figures and images for: Pericardial Disease in Patients with Cancer: Clinical Insights on Diagnosis and Treatment
Source: Cancers (Basel). 2024 Oct 12;16(20):3466. doi: 10.3390/cancers16203466 (PMC11505731; doi:10.3390/cancers16203466)

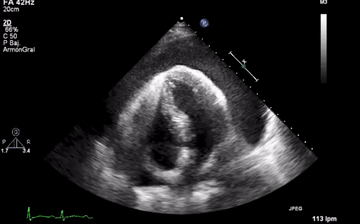

Supplement: Supplementary file 1 [file cancers-16-03466-s001.zip › cancers-3170220-supplementary.gif]
